# Observations upon the Character and Treatment of Lepoides

**Published:** 1840-02-08

**Authors:** William Harris


### MEDICAL EXAMINER.

DEVOTED TO MEDICINE, SURGERY, AND THE COLLATERAL SCIENCES.

No. 6.] PHILADELPHIA, SATURDAY, FEBRUARY 8, 1840. [Vol. III.

### OBSERVATIONS UPON THE CHARACTER AND TREATMENT OF LEPOIDES.

BY WILLIAM HARRIS, M. D.

(*Read before the Philadelphia Medical Society, and, since its discussion, somewhat modified.*)

The ancient medical writers were acquainted with this disease.

Hippocrates describes it under the caption of Ερπης εσθιομενης, and Celsus under that of *ignis sacer.*

Since their day, it has received different appellations from various authors, such as *noli me tangere*, *cancer lupus*, *formica conoswa*, *papula fera*, *ulcus tuberculosum*, *herpes exedens*, &c.

The phrase, *noli me tangere*, at an early period of surgical history, might have been adopted as a just warning against approaching this disease, as the surgeons at that time aggravated every case they attempted to cure. The appellation ought now, however, to be expunged, as, in modern times, a large number of cases have been radically cured in this country, and in Europe.

And *lupus*, although the term now in most common use, is certainly very inappropriate to the disease under consideration, as its slow destruction of flesh is quite opposed to the rapid manner in which the ravenous wolf devours its prey.

But, without attempting to criticise further the technical phrases that have been used to designate this disease, I will here declare a preference for that of *Lepoides*, an appellation given to it by Dr. Warren, of Boston.

Lepoides, from λεπος, bark, and ειδος, resemblance, better expresses the bark-like crust with which the early stage of most cases of this disease is characterized, than any term hitherto in use.

The face is the ordinary seat of this disease, and the nose is the point on which it commits its most frequent depredations. It assails, also, the cheeks, the temples, the forehead, the eyelids, the inner canthus of the eye, the lips, and the chin. Upon the trunk and limbs, too, it sometimes exercises its ravages. The front and back part of the neck, the shoulders, the breast, the neighbourhood of the articulations, the external face of the fore-arms, the back of the hands, and the top of the feet, are all occasionally visited by this frightful malady.

In most cases it attacks but one part of the body at a time; but, in some instances, it assails several regions of the same individual, either simultaneously or progressively.

The lepoides ordinarily commence as small, dark red pimples, or tubercles, which develop very slowly,—and in the commencement only the external layers of the derm are affected. Small, white, dry scales, are subsequently formed upon their summit, which, after an indefinite period, are thrown off, exposing a tender and inflamed cuticle, which gradually thickens, and from it exudes a viscid fluid, which is soon converted into a light brown crust. And in some cases crusts are, in this manner, cast off and renewed for several years before ulceration commences. In other cases, however, the casting off of the first crust is followed by ragged ulceration of the skin, which slowly involves the subjacent tissues, or destroys the surrounding derm, accordingly as it is of the *exedens* or *non-exedens* variety.

Occasionally, the crusts of several small tubercles unite and form a confluent scab, more or less extensive, but never painful.

I have seen two cases which appeared to commence in an obstruction of one of the excretory ducts of the sebaceous glands, seated in the substance of the derm. In each case the patient scratched away the obstruction with his nail, and squeezed out a cheese-like substance resembling a fine worm. This effort was several times repeated, and, after some days, an exudation took place, which hardened, and formed a small circumscribed crust, of a leaden colour, which afterwards terminated in a well-marked case of lepoides.

Bark-like crusts upon the face, however, do not always terminate in ulceration. In very old persons these crusts make their appearance in various parts of the face, resembling lepoides in appearance; but they are not accompanied with any suffering, nor do they ever terminate in sores.

The same kind of crusts sometimes form upon the summit of *moles* situated upon the face of aged persons, which, in earlier life, were smooth and healthy. Neither of these cases require any treatment.

Again, this disease sometimes appears in the form of a white elliptical tumour, more elevated in the centre than at the circumference, with a smooth, well defined margin, resembling an elevated cicatrix. Presently the surface becomes abraded, a crust is formed, and eventually a malignant ulcer follows.

Again, instead of the bark-like crust, there are cases which commence in a more prominent excrescence of a wart-like appearance. The wart, like the crust, is often cast off and reproduced before it terminates in ulceration. Afterwards the sore, in all its characteristics, is precisely like that which follows the casting off of the crusts. It is a genuine lepoides.

Again, there is a variety of the disease designated, by M. Biett, under the name of *lupus avec hypertrophie*, which is always connected with a scrofulous diathesis. This form of the disease is very rare in this country.

DIAGNOSIS.

*Noli me tangere*, *cancer lupus*, and *cancer of the skin*, are considered by several authors as different names for the same disease; but Rayer, in his "*Traité des Maladies de la Peau*," and Cazenave, in his article upon lupus, in the "*Dictionnaire de Médecine*," maintain that noli me tangere, and cancer of the skin, are synonymous terms,—but that lupus is the name of a distinct disease.

Cazenave states that the tubercle of noli me tangere, or of cancer of the skin, is solitary, while those of lupus are mostly in groups; that the tubercle of noli me tangere is the seat of acute, lancinating pains, while that of lupus is free from pain; that the tubercle of noli me tangere is confined to persons of advanced age, while that of lupus is rarely seen beyond the age of forty years; that in the noli me tangere tubercle, the surrounding superficial veins become varicose, while in the lupus tubercle no such enlargement takes place. Again, there is a difference in the ulceration. The fungus of the noli me tangere, or cancerous ulcer, is more elevated, and bleeds more freely when slightly touched, than that of lupus. The cancerous ulcer has its edges inverted, and is never covered with a dry, thick crust. The converse is true in lupus. To the above general rules, it is conceded, that there are many exceptions, and that before ulceration takes place the diagnosis is often very difficult.

The *lepoid* tubercles are sometimes distinguished with difficulty from those of *secondary syphilis*, (syphilide,) especially before ulceration takes place. It is worthy of remark, however, that the syphilitic tubercles are more numerous and of a copper colour, while the lepoid tubercle is of a dark red. The history of the case, too, will assist in the diagnosis.

After ulceration takes place, the difference between the two affections, however, is more marked. The ulcers which follow the syphilitic tubercles are deep, their edges tumefied, of a copper redness, and ragged; while the lepoid ulcer is of a dark red colour, and, if it be of the *non exedens* variety, occupies only the superficies of the derm. But if it be the ulceration which characterizes the *exedens* variety, the diagnosis is very difficult. Here, however, the history of the case will assist the practitioner in coming to a correct conclusion; and here, too, there is this decided difference, that the lepoid ulceration commences in the skin, and afterwards attacks, in regular succession, the subcellular tissue, the muscles, cartilages, and the bones,—while the syphilitic commences in the bones, and slowly extends to the derm.

CLASSIFICATION.

The disease under consideration has been variously classified by different authors; and that of Cazenave is the latest, and perhaps the best. He divides it into three varieties only. 1st. *Lupus qui détruit en surface.* 2d. *Lupus qui détruit en profondeur.* 3d. *Lupus avec hypertrophie.*

Those who wish to make themselves familiar with the appearance of the several varieties of this disease, may examine with advantage the coloured engravings of the splendid works of Alibert and Rayer, which may be found in the invaluable library of the Pennsylvania Hospital. For the privilege of examining these and other rare and costly works, I am indebted to the courtesy of the managers and librarian of that institution.

TREATMENT.

The remedies used in the treatment of this disease, may be divided, with propriety, into constitutional and local. And *arsenic*, if we regard the strong and respectable testimonials in its favour, has strong claims to be placed at the head of both these classes of remedies, as a number of well-marked cases of lepoides have yielded to the internal and external use of that remedy. *Fowler's solution of arsenic* is the officinal preparation which most authors recommend to be administered internally, in small doses—say, from six to eight drops, three times a day.

*Iodine*, too, if we regard the testimony of Lugol and others, is well adapted to the cure of this disease, as a constitutional remedy. The preparation which Lugol administers internally, consists of

| | |
|---|---|
| Iodine, | ℈ss |
| Potassæ Hydriodas, | ℈j |
| Aquæ Fluvialis, | ℥iiss |

Of which six drops may be given twice a day. This dose may be increased two drops every week.

The above is the formula in which iodine is administered in this city, and with great success, in the treatment of scrofula, and of glandular enlargements, especially in goitre, and in enlarged prostate.

Carmichael, of Dublin, reports five cases of wolf cancer of the face, cured by the internal use of *oxy-phosphate* and *sub-oxyphosphate of iron.* The same remedy was used at the same time as a local application. And Crawford, and others, have reported cures that were effected by the internal use of the *muriate of barytes.*

Various other *alteratives* are recommended by different authors, such as *l'huile animale de Dippel*, *la tissane de Feltz*, *la solution de Pearson*, &c.; but, in my judgment, the best alterative treatment consists in the administration of a three grain blue pill every night, at bed-time, and an ounce of the officinal compound syrup of sarsaparilla three times a day, or the same syrup containing two grains of corrosive sublimate to each bottle, in the same quantity, without the blue pill. The patient should, at the same time, be restricted to either a vegetable or milk diet, according to circumstances. And while I affirm that alteratives are valuable adjuvants in the treatment of this disease, I confess that my chief dependence is upon the local treatment, which consists in the complete removal of the diseased parts, either by the knife, or caustic. In the early stage of the disease, before ulceration commences, the preference of the patient might be consulted, as, in this stage, the one remedy has no decided advantage over the other. But, whilst admitting this, I maintain that, after ulceration has taken place, the caustic applications are entitled to a decided preference. In this stage, the knife, indeed, has never succeeded in a single instance that came under my own observations, while the caustic applications have, on the contrary, proved very successful remedies. The caustic not only removes the diseased parts, but it sets up a new and healthy action in the surrounding tissues, and the healing process consequently goes on very rapidly; but when the ulcerated parts are removed by the knife, the cut surfaces, instead of healing, sometimes put on the same ulcerated character that existed before the operation, and the disease now extends its ravages with greater rapidity than before.

In the early, or *crust* stage of the disease, if the patient prefers the knife, the operator may seize, with a small pair of narrow pointed forceps, the crust with about a line of the surrounding skin, and remove the whole with a single stroke of the scalpel, or with a pair of sharp scissors. The wound should be filled with lint and secured by a strap of adhesive plaster. Treating it now as a simple ulcer, it will generally heal in a week, or a fortnight, at most. In this stage of the disease, Burns recommends that a needle be passed quite below the base of the tumour, in the sound substance, and that a ligature be applied under this, so as to act on the healthy skin, and throw off all the diseased part. As soon as the slough comes away, simple treatment will ordinarily heal the ulcer.

In the second or ulcerated stage of the disease, various kinds of caustic applications have succeeded in effecting radical cures under the direction of different medical men, such as nitric and sulphuric acids, the lunar caustic, the actual cautery, muriate of mercury, preparations of arsenic, caustic potash, quick lime, &c.

Burns remarks, "there is nothing that I have ever tried that has disappointed me so seldom as the application recommended by M. Justamond, made by melting in a crucible, two parts of sulphuret of antimony and one of white oxide of arsenic. A single application of this remedy in form of powder to the ulcer, will cause a slough to come away after some days, when the ulcer that remains is to be treated with simple ointment." My brother, Dr. Thomas Harris, has repeatedly tried the above plan of treatment, as recommended by Justamond, and with complete success. He prefers this, indeed, to all other local applications in the treatment of lepoides.

M. Dupuytren had great confidence in the external use of arsenic in conjunction with calomel. He recommends that one grain of the white oxyde of arsenic be carefully combined with one hundred and ninety-nine of calomel. "If any crust covers the surface," says he, "let it be removed by a poultice, then sprinkle the sore with the above powder by means of a little puff. The sore puts on a healthy character and heals under this management. If the powder does not adhere well, it may be mixed with a little pulverized gum arabic, augmenting the quantity of arsenic." *La poudre arsénicale du frère Come.*

℞.—Arsenici Oxydum Album, ʒii

Sulphuretum Hydrargyri Rubrum, ʒiv

Sanguis Draconis, ʒiv

Misce.

It is directed that a sufficient quantity of the above powder to cover the ulcer, be moistened with a little water and applied in the form of a paste.

This, as a local application in the treatment of lepoides, is in high repute, at present, in the wards of M. Biett, *à l'hospital St. Louis*, an institution appropriated to the treatment of diseases of the skin.

*Warm baths*, *simple* and *medicated*, are extensively used, in that institution, in the treatment of lepoides and other diseases of the skin.

Arsenical pastes, and fluids in which arsenic is a prominent ingredient, have been completely successful in the treatment of many cases of lepoides; nevertheless, as their application has sometimes been followed by violent inflammation, and even the death of the patient, in consequence of the absorption of the poison, it becomes us to resort to them only in extreme cases, and then with great caution.

*The concentrated nitrous acid* is recommended by M. Averill, in preference to all other remedies, to be applied to the ulcer with a brush.

"*The red hot iron*," according to Dr. Macfarlane of Glascow, "has a powerful effect in changing the morbid action, producing healthy granulations and speedy cicatrization."

An extract made from the common poke berries, (Phytolacca decandria) has been applied to some well marked cases with complete success. Miss W., of Chester county, aged forty-five years, had a lepoides upon the side of her nose of ten years standing. At times it was covered with a crust, in extent perhaps half an inch, having a well defined red edge, with surrounding induration, and was accompanied with occasional shooting pains. Again, the crust would be thrown off, and the surface beneath would be partly inflamed and partly ulcerated. By the direction of a friend, she procured a quantity of poke berries, expressed their juice upon a pewter plate and allowed it to stand in the sun for several days until the more fluid parts were evaporated, when it assumed the consistence of an extract. This was spread upon a linen rag and applied to the diseased part twice a day, for a considerable length of time, when a complete cicatrix formed, and continued perfectly sound ever afterwards. This lady's grandmother had been cured, some years before, of the same disease, by the same remedy, under the direction of a *cancer doctor* of Lancaster county.

Another case of cancer of the face, in Chester county, in which the ulceration was unusually extensive, and of long standing, was cured by my friend, Dr. Latta, after a number of the ordinary remedies had failed, by a solution of Kreosote, in the proportion of four drops to the ounce of water, which was gradually increased to twice the quantity. Lint, saturated with this solution, was applied to the ulcer, and renewed twice a day. Under this treatment, the ulcers completely healed, and the cicatrices, up to this time, are perfectly sound.

Caustic potash, and quick lime, in equal parts, form, in my opinion, the most safe, and, when skilfully managed, the most effectual remedy in the treatment of lepoides.

My notes upon the two following cases, constituting two varieties of the disease, will be sufficient to show the precise mode of using it and its complete success.

Colonel **** ******* of the United States Army, in the autumn of 1838, on his way from Detroit to Florida, consulted me respecting an ulcer of two years standing, situated upon the left cheek just below the eye. Upon examination it proved to be a lepoides, and as he could not remain, at that time, more than twenty-four hours in Philadelphia, I directed him to dress it once a day with Griffith's adhesive plaster, spread upon black silk, and not to allow any empiric to touch it, as it was a malady easily aggravated by unskilful treatment. I advised him, moreover, to return to Philadelphia as soon as he could procure a furlough from the government, when, in all probability, in one or two months his disease could be radically cured. The colonel followed my directions implicitly, and returned to Philadelphia on the 17th of July last, and placed himself under my direction. Besides the ulcer on the left check, which was now about an inch in diameter, and of two years and a half standing, there was another upon the right cheek, which had made its appearance since I saw him last, about half an inch in diameter, and of three months continuance. These ulcers had both red, glairy, fungous surfaces, with edges hardened and slightly elevated.

The colonel stated that the first ulcer made its appearance in form of a pimple, the top of which he repeatedly scratched off, and squeezed it around the base until it began to ulcerate, after which he kept it covered continually with a piece of court plaster. And that the second made its appearance in form of a small red tubercle, the top of which he cut off with his razor while shaving; after which, it refused to heal, and continued an open ulcer, slowly extending itself. The complexion of the colonel was of a leaden hue, and his lips were of a blue or purple tinge. His habits were those of a gentleman of the old school. He ate a generous dinner and drank a good glass of wine every day, but never to excess.

In the commencement of my treatment, I directed him to lay aside his wine entirely; to avoid all heating exercises; to restrict his diet; to take a three grain *blue pill* every night; and to drink a bottle of Saratoga water in the course of the next day. These remedies were daily repeated, bringing away copious dark bilious evacuations.

On the 20th of July, I commenced the local treatment of the small ulcer, situated upon the right cheek, by taking a piece of common adhesive plaster, an inch and a half square, and cutting a circular hole in it, so large that its circumference would extend about a line beyond the circumference of the ulcer, to which I carefully adjusted it. I next took about ten grains of a powder consisting of equal parts of caustic potash and quick lime, to which I added one drop of water and made a paste, which I laid over the hole in the adhesive plaster, in contact with the exposed ulcer and its hardened margin. Next I covered the paste with another piece of adhesive plaster, a little larger than the first, and allowed the whole to remain undisturbed for fifteen minutes. After which the applications were removed, and a bread and milk poultice applied. During the fifteen minutes that the caustic paste remained in contact with the ulcer, our patient suffered intense pain, which subsided however so rapidly, after the poultice was applied, that in twenty minutes more he was quite easy.

The next day the ulcer and a line of its surrounding margin was quite black, which remained until the eighth day after the caustic was applied, when a slough came away about one-fourth of an inch in depth, under which was a healthy granulating ulcer. The poultice was now laid aside, and the ulcer dressed once a day in the following manner. First the ulcer was washed clean by means of a piece of soft sponge and a little castile soap and cold water, next a piece of lint wet with a strong decoction of white oak bark was applied, and over this a small piece of linen cambric spread with simple cerate, and the whole secured by a strap of adhesive plaster. This dressing was renewed daily, without any modification, until the ulcer was entirely healed, which was accomplished in twenty-two days after the treatment had been commenced.

On the 22d of July, I applied the same caustic paste, in the same manner, to the larger ulcer upon the colonel's left cheek, and allowed it to remain undisturbed twenty-nine minutes. The pain occasioned by this application was of a throbbing character, and so intensely severe that it was almost insupportable. It subsided, however, completely, in thirty minutes after the poultice was applied.

The slough from this ulcer was three-eighths of an inch deep, and came away upon the twelfth day, the granulations underneath being small and healthy. The subsequent treatment was the same as in the former case, and the ulcer continued to heal very kindly until the end of the third week, being reduced to about one-fourth the size it was when the slough came away, when the surface again put on a glairy, fungous appearance, and the healing process ceased. I now began to apprehend a failure, but determined to try the caustic potash in its crude state, and, accordingly the surface was touched with it slightly once a day, or once in two days, continuing, at the same time, the ordinary dressings. This plan of treatment was persevered in for ten days, without the slightest indication of the ulcer healing. I now decided to let the dressings remain three days undisturbed, by which the ulcer was diminished in size about one-half. It was again slightly touched with caustic, and a similar dressing allowed to remain three days more, and when this was removed, I had the gratification to see the ulcer completely cicatrized; so that on the 27th of August, thirty-six days after the commencement of its treatment, the second ulcer was completely healed. A piece of adhesive plaster was worn over the cicatrix of each ulcer for a week after they had healed, that they might become firm before they were exposed to the action of the atmosphere.

After the blue pill and Saratoga water had been continued a fortnight, they were laid aside, and a compound syrup of sarsaparilla, having two grains of corrosive sublimate to the bottle, was substituted; of which the patient took a fluid ounce three times a day. This was continued until the cure was accomplished.

It may not be improper to mention that, during the treatment, by my direction, Mr. Chiles, a skilful dentist of this city, removed from the colonel's mouth, several dead stumps of teeth. Whether they had any agency in the production of the ulcers I pretend not to decide; but in the treatment of all kinds of ulcers of the face, I have regard, invariably to the state of the teeth.

When the colonel left Philadelphia to resume his official duties, I directed him to abstain from wine and all stimulating drinks for at least one year, and at the same time, to live upon a diet less nutritious than his ordinary fare had hitherto been.

Nearly two months after his departure, I received from him a letter, dated "Detroit, October 5th, 1839," in which he says, "It gives me great pleasure to assure you that not the slightest symptom of a return of the disease upon my face has manifested itself since I last saw you in Philadelphia. The cure, I believe, is perfect, which has relieved me from much painful anxiety." The colonel is now in Florida, leading a very exposed life, but continues perfectly well.

CASE II.

Signor Don ****** ** ****, from Mexico, consulted me in October last, respecting a tubercle upon his nose, which was of an elliptical shape, the long diameter being about three-quarters of an inch in extent, and the short one about half an inch. Its situation was upon the left side of the nose, between the ala and inner canthus of the eye. This tubercle was white, smooth, and elevated above the surrounding parts about a quarter of an inch, so that if a Lima bean had been split in two, and the one-half laid with its flat surface in contact with the nose, it would have resembled it very much in extent and appearance. Although its general surface was smooth and glazed, there were several small holes at its lower edge, from which oozed, occasionally, a little viscid fluid, that would form a crust, which the patient would remove with his finger nail. Cases of lepoides, similar to this, are reported by Doctor John Burns, of Glasgow.

The preparatory constitutional treatment in this case was the same as in that of the case just related. And on the 7th of Novembr last, I commenced the topical treatment by cutting a hole in an oblong piece of adhesive plaster, of precisely the dimensions of the tubercle, which, when applied, fit closely around it. This was done in order to save all the surrounding skin, and to have as small a cicatrix as possible. The caustic application used in the former case was now applied and allowed to remain on about twenty minutes, which occasioned the patient very little suffering, and showed clearly that the tubercle was possessed of very little sensibility. The slough that was produced by the first application of the caustic did not include the whole tubercle, and I was obliged to make a second application, and subsequently to touch the edges several times with a stick of the pure caustic potash. The subsequent treatment, the same as in the former case, completely healed the ulcer in twenty-two days from the time the first caustic application was made, and the cicatrix is now so sound and healthy that I have no doubt the cure is radical.

In the commencement of the treatment of this case, had I cut the hole in the adhesive plaster sufficiently large to extend a line beyond the circumference of the tubercle, and had I allowed the first caustic application to remain thirty minutes instead of twenty, the tubercle would have been removed without any further effort.

The public mind has long been impressed with the belief that the empiric treats cancerous or malignant ulcers with more success than the regularly bred physician, and hence many patients, afflicted with these maladies, place themselves first under the care of an ignorant pretender, and in some cases fall a sacrifice to his unskilful treatment.

The empiric professes only to cure cancers which commence in the skin; but if the breast, the womb, the stomach, or the rectum be the seat of this fearful malady, what can the quack do? He can neither cure them, nor mitigate the patient's intense sufferings. But when he is consulted with regard to any tubercle, or ulcer about the face, he invariably pronounces the malady, however simple and easily cured, a cancer, and often acquires great reputation and large fees, not by his skill, but his tact or deception.

The regular practitioner is not always blameless of these results; for it too often happens that he neglects to study the character and treatment of the disease which has been the subject of these remarks, and when consulted, he rather shuns than courts its treatment, and thus throws the cases, which he might have cured, into the hands of the charlatan.

The works of Gibert, Rayer, Alibert, Cazanave and Schedel, Warren, Willan, Plumbe, Bateman, Burns, Cooper, and others, are full of valuable information with regard to this malignant disease, and the still better book of nature is always open to the study of the careful observer. Let the true physician, moreover, take care to distinguish himself from the false pretender, *with his secret remedies*, by publishing in full the circumstances and treatment of the cases which he has cured, and then not only will the profession be benefited and our science enlarged, but the public mind will be disabused, and the *advertising-nostrum-monger* reconsigned to his original obscurity.

